# Escaping the midway trap: A mixed method study highlighting roadblocks and coping mechanisms of female researchers in Cameroon

**DOI:** 10.1371/journal.pgph.0001759

**Published:** 2024-10-23

**Authors:** Nicole Fouda Mbarga, Sylviane Maffo Tsinda, Corinne Tchoula Mamiafo, Marcel Mbarga, Lamare Tchachoua, Julienne Ngo Likeng, Mireille Ndje Ndje, Emilienne Epee, Olga Bassong, Yap Boum, Sylvie Kwedi Nolna

**Affiliations:** 1 Women in Global Health (WGH) Cameroon, Yaounde, Cameroon; 2 World Health Organisation, Yaounde, Cameroon; 3 Catholic University of Central Africa, Yaounde, Cameroon; 4 Korea University, Seoul, South Korea; 5 University of Maroua, Maroua, Cameroon; 6 University of Yaounde 1, Yaounde, Cameroon; 7 Higher Women Consortium, Yaounde, Cameroon; 8 Epicentre, Yaounde, Cameroon; University of Washington Department of Global Health, UNITED STATES OF AMERICA

## Abstract

Across the world, women make most of the health workforce, but remain underrepresented in academia. This is even worst in Sub-Saharan Africa where women are the least represented as first and last authors in publications, especially in francophone countries. However, there is a lack of data on the reason explaining this inequity. Therefore, we sought to describe challenges which hinder the growth of Cameroonian female researchers and conducted a mixed method study over one year from January 2020 to December 2020. We included Cameroonian female researchers in health. For the quantitative arm, data was collected through an online google questionnaire. In-depth interviews were organized for the qualitative arm. Data was analyzed using python software version 21 for the quantitative arm and content analysis was performed for qualitative data. A total of 119 participants were included in this study. Most participants were aged between 25 and 35 years (72%) and they were Christians (94%). The majority had at least a PhD degree (29.6%) and they came from the West region of Cameroon (34.2%) the Northern regions were grossly underrepresented. More than half of our participants faced issues with balancing career, work, and academia and this is linked to culture. Socioeconomic, sociocultural, institutional, and environmental roadblocks hinder the progress and research productivity of most female researchers. The burden of unpaid maternity leaves, and sexual harassment remains high for most women. The use of English language as *lingua Franca* is perceived as another barrier for one on two women. However, there are coping strategies adopted by female scientists including the development of soft skills such as self-confidence, determination, and hard work. Extrinsic factors such as global policy, international partnerships, workshops, mentorship, and networking are also supporting women in global health. Gender-based interventions are critical to support women in escaping the midway trap.

## Introduction

Gender equity in science is a human right for women, and it is essential for sustainable development around the world [1]. However, the way forward to become a leader in science and academia is a tremendously challenging path for women globally and especially in Sub-Saharan Africa (SSA). Despite several efforts to advance women in science, around the world, there is a significant gender gap in the representation of women scientists who still represent only 33% of all researchers with broad disparities at national and regional levels [2]. In Southeast Europe and Central Asia, approximately one on two scientists is a woman [3].

The gender gap in science begins early on as only 30% of women are enrolled in the fields of Science, Technology and Engineering, and Mathematics (STEM) [3]. The number of women enrolling in the fields of STEM is still overall low (30%) [4], but even lower in SSA In General, underrepresentation of Africans in Science is alarming and African female participation in academia is even worst [5]. African women make only 30.4%, countries like Cameroon have only 21.8% of female researchers [4]. At the higher level of education in Africa, the gender gap gets further wide [5]. In Cameroon for instance, only 7% of professors are females [6]. Further evidence of this gap is the low proportional contribution of women to research, with fewer publications and trivial career growth [4,7]. Despite their willingness to participate in the transformation of African health care, women face several challenges where the social pressures of balancing work and family are stronger for women than for men [8]. Many women find themselves squeezed out of Science, Technology, Engineering and Mathematics (STEM) carriers because of several roadblocks [1]. The few women who successfully enter the STEM fields face many challenges that hinder career progress [9]. Moreover, women men comprised only 39% of first authors, 35% of last authors and 44% of single authors. The situation is worst in SSA as Women from South Africa and Nigeria publish more than those from other SSA countries, though women represented at least 20% of last authors in 25 SSA countries [10]. Although there has been some improvement, women rarely take the last author position as it is most often reserved for a male principal investigator [11]. Unfortunately, this phenomenon is rarely described in the countries of sub-Saharan Africa especially in Francophone countries such as Cameroon.

African challenges have several scientific solutions and broadening the participation of Africans in Science and unleashing the potential of women is critical. Closing the gender gap in science begins by documenting the contextual challenges and opportunities for women. This study seeks to describe the challenges and local solutions to women researchers in global health, thereby producing evidence to inform policy making on gender issues for African women in STEM and develop gender-based interventions to increase equity in global health.

## Materials and methods

The study was conducted over a period of one year from January 2020 to December 2020. Study participants were Cameroonian female researchers in the field of health across the country. In the context of this study, a researcher in the field of health was any professional or student involved in the constitution of new knowledge, new facts, or evidence in the field of health or allied professions with tangible evidence including a study protocol, a thesis, a peer-reviewed article and/or a book [12].

We performed a mixed methods study with a cross-sectional study and in-depth face-to-face interviews to acquire general and in-depth knowledge and provide a synoptic understanding of challenges and opportunities of Cameroonian women researchers in health. We used a sequential explanatory mixed methods design to confirm and further explore the results from a quantitative data collection and analysis followed by qualitative interviews and interpretation to further explore quantitative findings [13].

### Quantitative methods

This first phase provided knowledge of to what extent women researchers in health face challenges and benefit from opportunities. Additionally, the quantitative phase provided a pool for the selection of participants for the qualitative follow-up interviews.

We used targeted networks of females in global health to identify potential Cameroonian women researchers or groups of women researchers. To obtain a comprehensive set of participants, we targeted various networks of female health professionals including the Higher Women Consortium, the Women in Global Health Cameroon, the Cameroonian Association of female medical doctors and the Organization for Women in Science for the Developing World (OWSD) Cameroon chapters. These associations have as members women who have at least been initiated into research and in particular, the HIGHER Women Consortium was identified as the main potential target for recruiting study participants. HIGHER Women Consortium is a Cameroon-based consortium is designed to provide the country’s early-career women scientists with the mentoring, skills development, and career planning they need to establish an enduring presence in the field of health research.

HIGHER Women Consortium is a consortium of women’s health researchers at various career levels, both mentors and mentors / proteges [14]. The consortium encompasses several women health researchers, including senior and early career researchers. We also used professional and social media platforms to identify more research groups. Cameroonian researchers were recruited through their institutional web-based mailing lists and recommendations from their colleagues. Since it was not possible to have the exact number of Cameroonian women researchers in the field of health, we considered the total number of women who joined the HIGHER association to calculate our sample (i.e., 170 women) using the Lorentz formula. The minimum sample size X was therefore 119 women.

An online survey was carried out through a questionnaire designed on google form in both French and English. Based on previous studies describing the challenges faced by women in global health and academia [8,15,16] the challenges of women researchers were grouped into 3: socioeconomic challenges, environmental challenges and institutional challenges related to sexual harassment and discrimination. Cameroon is a bilingual country with French and English being the official languages but very few Cameroonians speak both languages. Most Cameroonians are French speakers [17]. Since English dominates in scientific journals [18], the English language could constitute an obstacle for a French-speaking researcher in Cameroon. As such, language barriers, access to information and sources of funding were seen as subgroups of challenges in the research environment. The last section of the questionnaire assessed coping mechanisms suggested or used by Cameroonian female researchers.

We conducted a pre-survey to test the questionnaire and to improve on the quality of the assessment. The information collected during the pre-test made it possible to estimate the response time at around 20 minutes. After validation of the questionnaire, it was shared to various female’s platforms on WhatsApp, LinkedIn, Twitter and by electronic mailing. The online questionnaire, once completed anonymously, was recorded and data was automatically available for downloads as an excel spreadsheet. The data was analyzed using Python software version 3.12.0. Univariate analysis was performed, and variables were summarized as counts and percentages, means and median where appropriate.

### Qualitative methods

The second, qualitative phase, contributed to a deeper understanding of roadblocks experienced by female researchers in health as well as coping mechanisms. To ensure reliability, an interview guide was designed to consistently focus on the following main themes: obstacles to career growth in research and in-house solutions. We performed a nested sequential sampling, selecting key informants at various professional levels (early, middle, late) for the qualitative phase from those who participated in the quantitative sample. The sample size of 6 participants was considered sufficient to reach saturation [19].

The first and the second authors conducted single face-to-face interviews with key informants at various professional levels. In-depth interviews were organized as follows: introduction of the investigators, provision of general information about the study and voluntary signing of consent to participate in the study. The interviewees were informed about local customs and cultural norms and were fluent in English and/or French. Interviews were conducted in French or English, depending on the participant’s preferences. The interviews were audio-recorded and then translated and transcribed into French or English by two investigators. To ensure the best possible quality of the study data, they were transcribed within 72 hours of the end of the interviews. For confidentiality purposes the women were attributed anonymous names. We performed deductive qualitative content analysis using themes identified through literature review [8,15,16]. Interview transcripts were reviewed to determine content relating to barriers to career growth in research as well as proposed solutions. A coding system was developed to represent common topics encountered while reviewing the transcripts. Each interview was read several times to identify units relevant to information about challenges and opportunities of female researchers. These units were condensed and coded to identify the manifest content of the text. Codes that shared a commonality were organized into 4 categories: socioeconomic challenges, environmental challenges and institutional challenges related to sexual harassment and discrimination as well as coping mechanisms.

### Ethical oversight

The study was approved by the Institutional Research Ethics Committee for Human Health (CEIRSH) of the School of health Sciences of the Catholic University of Central Africa 2020/020103 / CEIRSH / ESS / MSP. All study participants gave a written consent documented in the online google form (quantitative arm) and/or a verbal consent to participate in the study.

### Participant and public involvement

We launched open calls through social media for contributions to the survey. The research question was set to address the challenges and coping strategies of female researchers in health, and the design was based on ongoing engagement with the Higher women consortium and our understanding of how to engage and gain their involvement most effectively. The study was entirely open throughout all the steps and the time taken to complete the survey and taking part in the in-depth interviews was made clear to participants.

## Results

### Questionnaire survey

A total of 119 Cameroonian female researchers in health participated in our study and most of them resided in Cameroon (84%). Many of these women belonged to the age group (25–35] (72%), they were Christians (94%) and they came from the West region of Cameroon (34.2%) while the Far North of the country was underrepresented (0.9%), the North, Adamaoua and the South regions of Cameroon were not represented in the study. There was a high preponderance of married women (45.7%) with at least one to three kids (37%). Among women who had the privilege to get a maternity, only 27.2% had a paid maternity leave. Most study participants had a *PhD* (29.6%) and fewer participants had a full professorship (2.5%). The most common title used by participant was “Miss” (35.2%). A great deal of participants was affiliated to a university (50.4%), with a hospital (public/private) 20.9% but the majority had less than 02 years of professional experience (34.7%). Participants with more than 10 years of experience were underrepresented (13%). A bulk of women had no international peer-reviewed publication (51.5%) and no publication as first (49%) and last author (79%). Most participants had never received international grants (69.4) nor awards (79.5) (Table 1).

**Table 1:** Sociodemographic characteristics of females in the questionnaire survey (n = 105).

Variable	Category	n (%)
**Age group**	<25	19 (15.9)
	25–35	73 (61.4)
	36–45	20 (16.8)
	46–55	7 (5.9)
	Mean SD	
**Region of origin**	Centre	13 (12.3)
	Littoral	9 (8.5)
	West	36 (34.2)
	South-West	12 (11.4)
	North-West	30 (28.5)
	East	3 (2.8)
	South	2 (1.9)
	North	0 (0)
	Far North	1 (0.9)
	Adamaoua	0 (0)
**Religion**	Christian	100 (97.2)
	Muslim	1 (0.9)
	Others	2 (1.9)
**Level of education**	Bachelor’s level	22 (20.9)
	Master’s level	21 (20)
	PhD	33 (31.5)
	Doctor of Medicine (MD)/ Pharm D	28 (26.7)
	Full Professor	3 (2.8)
**Title**	Miss	37 (35.2)
	Mrs	24 (22.8)
	Dr	37 (35.2)
	Pr	7 (6.6)
**Matrimonial status**	Married	48 (45.7)
	Single	41 (39.1)
	In a relationship	12 (11.4)
	Widow	1 (0.9)
	Divorced	3 (2.8)
**Preferred language**	English	52 (44)
	French	42 (35.5)
	English and French	24 (20.3)
**Affiliation**	University (Public/ private)	53 (50.4)
	Hospital (Public/ private)	22 (20.9)
	Other public services	12 (11.4)
	Non-governmental organisations (NGOs)	20 (19.1)
	Commercial research organisation	3 (2.8)
	Pharmaceutical company	1 (0.9)
	Other	5 (4.7)
**Years of experience**	0–2	40 (34.7)
	3–5	32 (27.8)
	6–10	28 (24.4)
	>10	15 (13.1)
**Number of kids**	0	37 (37.3)
	1–5	59 (59.6)
	>5	3 (3.1)
**Paid maternity leave**	Yes	24 (27.2)
	No	64 (72%)
**Number of publications as first author**	0	50 (49)
	1–5	38 (37.2)
	>5	14 (13.7)
**Number of publications as last author**	0	71 (79)
	1–5	15 (16.8)
	>5	6 (6.7)
**Number of international peer-reviewed publications**	0	51 (51.5)
	1–5	21 (21.2)
	>5	27 (27.2)
**Number of national peer-reviewed publications**	0	62 (69.6)
	1–5	27 30.4)
	>5	0 (0)
**International grants or funding**	0	59 (69.4)
	>1	26 (30.6)
**National grants or funding**	0	68 (85)
	>1	12 (15)
**International awards**	0	66 (79.5)
	>1	17 (20.4)

**In-depth interviews,** three categories emerged in the interview data including sociocultural, institutional, and environmental challenges and opportunities for development. Table 2 presents characteristics of the participants in the in-depth inter-views.

**Table 2:** Characteristics of participants in the in-depth interviews (n = 5).

Pseudonym	Age (years)	Profession	Educational level	Marital status	Region of origin	Years of professional experience	Number of kids
**Participant 1**	30	Medical Doctor	MD	Married	West	06	01
**Participant 2**	40	High official at MOH		Married	West	14	04
**Participant 3**	41	High official at MOH		In a relationship	Centre	16	02
**Participant 4**	24	Epidémiologist		In a relationship	West	02	01
**Participant 5**	40	Psychologist		Single	West	08	01
**Participant 6**	51	Clinical researcher	PhD	Married	Littoral	20	04

*MOH Ministry of health.

### Socio-economic and cultural barriers of women health researchers

Most women researchers (37%) devoted less than 20% of their time to research and the remaining time to household chores for domestic duties and household chores. Unfortunately, most women (63%) could not afford a household help leading to one of seven women (14%) researchers feeling tired almost all the time. However, several participants receive various forms of family support: psychological (32.8%), physical support for domestic duties (24.4%) and financial (16%). It is worth mentioning that more than half of these participants (52.1%) felt negatively judged by their neighborhood concerning the way they balance career, family, and academia. A proportion of 15% of participants mentioned that they lose one out of two opportunities for career or academic development due to the burden of domestic duties and social pressure.

Women researchers interviewed described in details socioeconomic pressure they face as they try to balance career, academia, and family. The women interviewed mostly lived with their families, and devoted an average of 4 hours of time daily to domestic and family chores; as we can confirm in the words of respondent 01: *“Well… I would say that… If we stick to the fact that the work ends at 4 p*.*m*., *I will say 4 hours*, *from 4 p*.*m*. *to 8 p*.*m*. *because after that*, (…)”. In addition, most of them devoted around 2 to 5 hours of their time daily to research. However, this is not fixed, depending on moods, this time decreases or increases, consuming the entire day of the researcher. This is supported by the statements of respondent 4, who said to this effect: *“On average 01h30 per day; But there are days when if I go beyond that*, *there are days when I am galvanized*, *I can even spend a whole day researching; and days when I don’t even feel like doing the research*.*"*; and from respondent 02: *“With the evolution of technology where we have computers*, *telephones and internet connections; I am constantly doing research even when I am in the kitchen”*. And most being in the family, the support at home comes from this family, restricted or extended, and even from third parties like a nanny. It all depends on the organization, as respondent 02 supports: *“I organize myself with the children and my husband that is*, *the restricted family*. *Everyone has a task they to do*, *and I coordinate the rest*. *I also do the hardest so as not to leave the load on the children*. *We support each other and it doesn’t weigh too much on a person”*. In the same line, respondent 04’s statements maintained that the family removes a moral burden: “(…) *I have a nanny; when I’m not there*, *she takes care of the child all day*. *Apart from that*, *there is also his father*, *and his grandmothers*. *So*, *on that side*, *I have no worries*.*”*

However, research also encroaches on the personal life of women scientists, and it is a burden on their relationship with their partners, husbands, children, and friends. Indeed, research is time-consuming, and this affects their social relations. This can be confirmed through the statements of respondent 01: *“Hmmm…I would say that sometimes the partner complains that I don’t have enough time for him*, *so I would say that the burden of a researcher who is equally employed it’s really the lack of time…in fact you don’t have enough time to devote to anything else other than your job”*; and respondent 03: *“(…) If we take 8 a*.*m*. *to 6 p*.*m*., *regular work maybe done between 8 a*.*m*. *to 3 p*.*m*., *and the rest of the time; it is research*. *And when there are off-peak hours*, *it’s always research*. *For example*, *during the COVID-19 era*, *sometimes I had to close at 10 p*.*m*. *then go home*. *So*, *it seriously encroaches* (…) “. In addition, most female scientists have lost professional development opportunities such as scholarships, travel opportunities, jobs and even opportunities for social development because they bear the weight of the family, as respondent 01 stipulated: *“Hmmm… Yes*! *I have already had to lose at least three travel opportunities*, *two training opportunities*, *a scholarship*, *and even employments and I got married late (…)”*; and respondent 05 confirmed that: *“Yes*, *yes*, *yes*! *At a certain point*, *I stayed behind because of the weight of the family; I worked to the point of losing a pregnancy*! *"*.

Furthermore, in some African cultures, the intellectual woman is perceived as a rebellious, proud, stubborn woman as respondent 02 said: *“(…) in Africa*, *the intellectual woman is usually referred to as emancipated*. *And in our cultures*, *we don’t like emancipated women*. *We often say that they know too much*, *therefore they will no longer be submissive in homes*. *They will no longer even be subject to the elders*. *(…) Although elsewhere it is rather the opposite where people would even like to have an emancipated woman in the family; because we know that it will give a better image of the family*. *But in our context*, *educated women are taken for rebellious women*, *and even prostitutes…”*. Concerning availability of female scientists in family, most of them are unavailable to assist their loved ones. Example, respondent 01 argued that: *"I hardly participate in anything anymore*, *yes*! *Unless it has to do with financial assistance*, *(…)*, *but physical assistance*, *no*!*"* Although they are mostly unavailable, some women researchers seem to integrate easily into their community. This is confirmed by the statements of respondent 02: *"No problem*. *The community has nothing to do with the career*, *huh*! *The community is the behavior we have towards others*. *Yes*! *If you are a good person*, *you will be with others; if you are a bad person*, *whether you are a researcher or not*, *it will show up”*.

Regarding cultures and beliefs, responder affirmed that: *“even in ancient cultures*, *we saw that when a parent had two children and he did not have enough money to send both to school*, *he said*, *the female child stays in the kitchen and helps with domestic work*. *The chance is given to the male child to the man*, *because he knows that he can do great studies*, *and then feed the whole family*.*”*

### Institutional challenges

In addition, an important proportion of women (42%) is worried about the impact of research on their social life especially marriage proposals. One out of two women have taken career breaks to take care of kids or to get a balance between personal life, career, and academia. One of four women get to take a maternity leave during doctoral studies or employment however, and among these women only one of three benefits from paid maternity leave. Among women researchers requiring a maternity leave in the course of an employment of studies, three of four women could not take a maternity leave as they feared losing their job or getting dismissed from their study program.

Approximately one out of two women (54%) has been harassed, humiliated, or intimidated during her research path or career while four out of ten women have received attacks on their dignity, to advance their career / or research. For many women (39%), this has led to negative consequences in the work environment for refusing to engage in sexual behavior. Unwelcome sexual advances, offensive sexual remarks or discriminatory remarks are common in the day-to-day life of some female researchers (44.6%) and approximately half (49.6) of these participants are scowled at and criticized because of their gender. All study participants felt that they had to do extra-work of varying extend to receive a recognition. In decision-making discussions at the workplace, 37.8% of women are expected to remain silent and not contribute. Most women (38.7%) work in an environment where males represent the majority at the top leadership.

Overall, it also appeared from in-depth interviews that Cameroonian women researchers face sexual harassment and discrimination in the workplace. There are several types of sexist stereotypes. Concerning that, respondent 02 said: *“Okay*! *Without citing contemporaries*, *I was harassed during the writing of one of my master’s dissertation whose title I will not make mention of*, *Yes; Because it can easily be recognized*. *So*, *I was really the victim of harassment with the supervisor*, *(…)*. *A female scientist sometimes pays for her research sexually*, *financially; that must be said*. *There are very few who even have the chance*, *I’m going to say like that*, *who get through without spending anything*, *and unfortunately*, *that’s how it sometimes works in our context*”. And respondent 03 said: “Most of the time in professional circles, women are harassed and bullied *“If you are not with me*, *you lose your benefits*! *"*. *The superior on you*! *(…)”*

Respondent 01 affirmed that: *"Speaking of sexist stereotypes in the professional environment*, *one has the impression that women evolve perhaps because some have special and privileged relationships with male senior officers* ". Moreover, these women participate in decision-making in the same way as their male counterparts, and have the same opportunities for development, although conditioned by their dynamism, without distinction of sex as confirmed by respondent 01: *“They participate if they are competent*! *"*. Speaking of development opportunities in some instances in Cameroon, respondent 01 said: *“When there is an opportunity to upgrade people*, *women are favored*. *When a woman is competent*, *she is put first on the list”*.

### Environmental challenges

In addition, a plethora of women face difficulties to obtain scholarships and opportunities for development (54.6) as well as significant difficulties to access research materials (56.3%). English language is a barrier for as much as 47% of female researchers in health. All women researchers felt unsupported by the government of Cameroon in their research paths. One of ten women receive significant local financial support.

In-depth interviews also confirmed that women evolve in strenuous research environments. Women researchers encounter difficulties in obtaining grants, access to information and publication, as well as government support. Indeed, for the difficulties of obtaining scholarships, respondent 01 affirmed that: *“Most often there are opportunities and people don’t tell you*. *First there is communication*, *then; well…*, *that’s mostly it*! *It’s mostly communication*. *Because people know that if you are aware of the opportunity and your file is good; you are competent; your case will get it*.*”*. The following statements also support the evidence of the difficulties of access to information, which circulates in restricted groups: *“They like to hide that*. *As we like to call their ’clergy’*. *It’s between the clergy*, *you’re not concerned*.*”*, (Respondent 01). In the same line, respondent 03 said: “*No; it’s not easy*, *it’s not easy*. *You often must juggle to access certain types of information*. ". The difficulty of publishing often appears to be linked to access to information: “(…) *I missed this chance because I had missed the information once*. *The news was sent over the internet concerning opportunities for publication*, *and during that period*, *the connection was unstable*. *I saw the news so late*, *and the deadline had passed*” (Respondent 02). And sometimes these publication difficulties can have other origins, such as the position of the female researcher in the paper: “*For publication*, *I am in the process of publication*. *But*, *since my work is with a Director*, *I think he will put his name first*. *Yet I was the one who was on the ground*. ".

About government support, women researchers maintain that it is much more from international partnerships that they receive support: *"The scholarships that I obtained were allocated to me by international institutions*. *So it is not the Cameroonian government… (sighing) speaking of government support*, *I haven’t really had that*. *Yes*, *the scholarships to attend my master’s program*, *were from the AUF with a trip out of the country*, *I also went to Portugal with the support of an organization engaged in the fight against Parkinson disease*. *All of these are international structures”*; and these facts were confirmed by (respondent 02, respondent 03): *“Of the government*? *…No*! *(…)” “UNICEF rather contributed to professional development”*.

### Opportunities of career and academic growth for women health researchers

In terms of opportunities for growth, Dialogues with renown scientists as well as workshops appears to play a significant role, contributing to the growth of 3 on 4 participants. To a lesser extent, international partnerships, networking, and mentorship also represent opportunities for academic and professional development for female health researchers.

Concerning mentorship, women health researchers appreciate a little more female mentorship (55.5%) when compared with male mentorship (51.2%). Regarding areas of female mentor support, female mentors provide support in skills development (39.5%), career-personal life balance (10.9%), networking / lobbying (9.2%) and goal setting / planning career (3.4%). In fact, 35.3% of female researchers admitted that if they had mentorship opportunities, financial support, fixed working hours, or family assistance they would not have had to take a career break.

Women interviewed confirmed these findings and they gave an account of some opportunities for growth and coping mechanisms to challenge the status quo and coping strategies for growth and development.

### Development of soft skills

Self-motivation, self-confidence, faith and hard work are key argued respondent 02: *"I can’t say that I really succeeded*, *but I know that I am on a good career path*. *Unlike some people*, *I got to a certain level*, *and I evolved while I worked*… *You have to believe in what you do*! *You must be determined when you pursue your goals*…*It’s true that it’s difficult*, *but you must set your goal*, *pursue it until you reach it…You must always put God at the center of everything you want to do in life*, *because he directs your life; guide your search*. *And*, *you don’t have to be a lazy woman*, *you must be hardworking*. *You must be driven by a spirit of dynamism and ambition; you must have a push for reading; And you even ought to surpass yourself at certain times…Most women who have succeeded in life*, *if you have to look at their history*, *you will see that somewhere they surpassed themselves*. *When you are a woman in our context*, *you are already disadvantaged*, *in this case you have to make millions of efforts to succeed in reaching a level*.*”*

About soft skills respondent 04 said: *“I think the success factors are determination and dedication*, *because when I decide to do something*, *I am very dedicated to it*. *Until I reach the goal*, *I don’t stop*. *And my dreams push me every day to go forward*, *to be able to achieve my goals*. *That’s all*!*”*, and for respondent 05: *“Determination and we get to focus a lot more on the objectives we are set to achieve*.*”* For respondent 03, being ambitious is critical: “(…) *You always have to stand out from the others*, *whether they are males or other females*. *Otherwise*, *your added value is not seen…I am a researcher*, *I research in my field; I will publish; I will do this; I’m going to do that*, *and I stick to that*. *But when you say*, *“No*! *I want to be recognized*, *I have ambition*, *I want this… you have to give more…in addition to doing what you love to do…because you want recognition”*.

On the other hand, women researchers most often use coping mechanisms such as observation, listening, sociability, and mutual respect in their professional environment. This is confirmed through the words of respondent 01: *“When I arrive in an environment*, *I observe for a long time*. *I speak very little; I study everyone’s behaviors and I start to adapt just to their behaviors*. *You must observe a lot*, *talk less*, *because what also kills women a lot in the workplace is useless chats »;* or again, from respondent 03: *“I am very sociable*, *very cheerful*! *And I bring this joy wherever I am (while smiling*, *speaking to his colleague nearby*, *who moreover confirmed his statement)”*.

### Mentorship

In the qualitative arm of the study, female scientists preferred male mentors when compared to female mentoring as confirmed by respondent 03: *“Okay*, *I’m going to go down to earth*. *Men mostly (pause) work down to the detail*. *And when they go to criticism*, *it’s without complex*, *straight to the point*. *On the other hand*, *you must deal with the moods of female mentors*. *There are some who may not even provide proper mentorship often out of jealousy*!*”*. Both male and female mentorship are not perfect though. For respondent 03: *“a disadvantage of male mentoring is earlier as we said*, *he may be in love with you*. *I faced that and since they couldn’t give me someone else as a mentor*, *I preferred to give up*. *Another downside of having a woman as a mentor too is that…the woman will hardly go to defend you*, *yet when it is a man*, *he goes there frankly (nodding his head)*. *So*, *each has its pros and cons*. *But if it was up to me to choose*, *because when you go beyond the stage of falling in love; it really works*! ".

### Global policy

Moreover, the simple fact that women researchers are “women” represents an opportunity for them. In some cases, female applications are encouraged as respondent 02 said: *“On the other hand too*, *there is something that now wants more female applications to be given priority*. *So*, *on the other hand also*, *as we know that the woman is often less privileged compared to the man”*.

## Discussion

We found that that most of the women researchers in global health face several challenges and they find themselves trapped in the midway of their career. These include socio-economic challenges including difficulties to balance career, academia, marriage, and childcare; and these challenges are linked to the culture that attributes a predefined status to women. The researcher’s professional environment is hostile with limited national support, and they face institutional challenges that are linked to stereotypes, discrimination, prejudices, and language barrier. Most women in this study have limited research productivity (publications, research grants and awards). Nonetheless there are existing coping strategies for growth including development of soft skills, international partnerships, workshops, mentorship, and networking. The qualitative analysis is aligned with quantitative results and, while showing that soft skills and global policy play a significant role in women academic and career growth.

In the current study, we found that many female researchers feel the double pain of having a family and being a researcher, made worst by sociocultural norms. Having a paid maternity leave is still a privileged for some women researchers in Cameroon. According to the Cameroon labour code, female employees are entitled to 14 weeks of paid maternity leave which can be extended to 6 weeks in case of proven illness [20]. However, some women get back to work earlier due to financial constraints or the fear of losing their job. Unfortunately, in Africa, there are discrepancies in protection of female employees during maternity and this affects mother’ and child ‘health [21–23] thereby limiting productivity of these women [24]. In fact, our study highlights tremendous roadblocks to female authorship in science. These findings are consistent with those of Giannos et al (2023) showing marked female underrepresentation in first and last authorship over a ten-year analysis of scientific papers despite continuous increase [25]. It has recently been observed that Scientific African women publish less because of the burden of household duties, restricted mobility and teaching [26]. These challenges could contribute to female underrepresentation in research awards and grants [27] and likely worst for African women.

Although there has been some progress over the past decades, gender gap in STEM is prominent, many researchers have focused on this subject to understand the problem in the quest for solutions [21,28]. There are so many challenges that hinder the participation of females in STEM thereby affecting the growth of the few female scientists available. Along the lines, UNESCO reports have raised alarms about several factors preventing females from progressing and participating fully in science. Some roadblocks include household and care responsibilities, early marriages and unwanted pregnancies, cultural norms favoring boys and gender-based violence in the school environment [3,29,30]. For Sougou *et* al, family and environmental factors represent the greatest challenges to the full participation and progression of female scientists [31]. Many women struggle to balance work and family, Eccles *et* al, even argued that for the sake of family, women tend to make more occupational sacrifices than their male counterparts [32]. As such, several women tend to think academic careers are not suitable for women with family goals [33]. This hinders the growth of women and leads to underrepresentation of females among full-time associate professors and full professor faculty positions [34]. The participation of female scientists in this study confirms these findings with full professor representing only 2.5% of participants.

Social norms and cultural beliefs also hinder the growth of female researchers. Our study sample had no participant from the Northern regions of the country. According to reports from the Cameroon Central Bureau of the Census and Population Studies (BUCREP), the Far North, the North and Adamaoua are regions in Cameroon where are found the least educated people [35]. Still according to the BUCREP, education rates are lowest in poor households and the Northern regions have the highest indicators of gender discrimination in the country [35]. This could explain disparities in the representation of female scientists from the Northern region of the country in the current study. In addition, in these Northern regions of the country, it has been observed that the choice to enroll one child rather than another is motivated by gender with boys being privileged to the detriment of girls [36]. Furthermore, early marriage is another social phenomenon which justifies the low rate of education, particularly among young girls in the Northern regions of Cameroon. From the age of thirteen, the teenager is considered mature enough to get into marriage [36]. In the collective consciousness, it is accepted that the vigor and intelligence of children is inversely proportional to the age of the mother. Prolonged schooling thus appears as a brake on the affirmation of the femininity of the young girl to give life [29].

Our findings confirm that institutional factors such as harassment, discrimination and prejudice also contribute to the underrepresentation of female scientists at the higher level. Wang and Degol described the existence of unexpressed or unconscious discrimination against women at the higher education level [37]. Stereotyping is an old phenomenon, and the gender male has been associated for so long with higher positions within organizations as results of authority and competence. Stereotypes appear to be imbedded in behaviors early from childhood and constitute a major hindrance to the representation of women in higher level managerial positions in organizations or in academia [38]. Gupta *et al* described the triple burden of female scientists including: the pain of being in organizational environments with stereotyped norms, the burden of bearing unequal domestic work and relative exclusion for powerful networks [39,40] which we also observed in our study population.

On the other hand, sexual harassment was found to be common for half of our study participants. In a similar multicentric study across 55 countries in the world, Fathima and collaborators reported workplace sexual harassment in one of four female scientists [2]. According to the International Labor Organization (ILO), sexual harassment takes the form of a quid pro quo or working in a hostile environment, and it is usually grossly underreported. Sexual harassment weakens relationships at the workplace and hinders productivity. Thus, in our context, half of female researchers would suffer from an impaired productivity because of hostile environments dominated by males. ILO proscribes sexual harassment and discrimination on the grounds of maternity and family responsibilities [41]. Unfortunately, Cameroon has not yet ratified the Violence and Harassment Convention, 2019 (No. 190) which was enforced on the 25^th^ of June 2021.This makes it challenging to prevent and address gender-based violence and sexual harassment in the workplace in Cameroon [42].

Another huge environmental barrier which threatens the advancement of one in three female health researchers in Cameroon is the language barrier. Unfortunately, the hegemony of English in Science and global health continues to be felt although the world most spoken languages are Chinese, English, Hindi, Spanish, Arabic and Portuguese [43]. Worldwide, most articles are published in English contributing to the segregation, invisibility of several scientists and the fruitlessness of their work [44,45]. This is especially true for scientists in francophone countries like Cameroon. The use of English language as the main scientific language thus perpetrates inequalities in science and this is true for scientists in Countries like Cameroon where most educated people speak French [36].

Intrinsic coping mechanisms included the development of soft skills (self-motivation, self-confidence, determination, faith and hard work) while extrinsic factors were international partnerships, global policies favoring gender equity, workshops, mentoring and networking. Mentoring by African women provides concrete role models that can be emulated and thereby facilitate growth and emergence in women researcher’s careers [46].

These intrinsic coping strategies are in-line with those described by Fathima and collaborators in their multicentric study [2]. Other coping mechanisms reported by Fathima *et al* included recreational activities such as relaxation, hobbies, exercise, sleeping [2]. Extrinsic factors described by Fathima et al that were slightly different from ours included childcare support and workplace support which are quite limited in our setting. Regarding coping mechanisms, it appears that Fathima’s cohort followed relied more on or intrinsic factors than extrinsic factors [2].

One of the strengths of this study is that mixed methods were used to deepen the understanding of challenges and coping mechanisms of female researchers in health. Despite the originality of the current study, our findings should be interpreted considering the following limitations. Firstly, participants were Cameroonian female researchers in health who were invited to participate in the study based on personal contacts and networks of the investigators and a snow balling process. As such, the sample selection was non-random, and this could limit the external validity of the findings. However, the study participants were recruited from the main women researchers’ associations in Cameroon. Therefore, our findings give us some pointers towards the major challenges experienced by female scientists and the coping strategies they adopt. Secondly, the sample does not have representation from all ten regions of Cameroon, the Far North, the North, Adamaoua and the South regions were not represented in the study. In addition, the distribution was not proportionally allocated based on population size, resulting in greater representation from some regions and under representation from others. Therefore, the findings of our study may not apply to any specific region of Cameroon though the low representation of other region could be an indicator of the heterogeneity of researcher in our country. Finally, the study was limited to female health researchers, therefore the generalization of findings to another field is challenging. Our different hypotheses seem to be insufficient to explore the challenges faced by Cameroonian researchers. The challenges mentioned in our study cannot explain all the obstacles that women health researchers face in general.

## Conclusion

In sum, most women researchers in Cameroon are trapped midway with limited academic productivity. Domestic and work-related stress resulting in poor work-life balance, sociocultural beliefs, institutional challenges including sexual harassment, stereotypes and language barriers experienced by the Cameroonian female scientists hinder their growth. Intrinsic factors like self-motivation, confidence, and dedication, as well as extrinsic institutional factors like international partnerships, workshops, networking, mentorship, and female friendly management policies are coping mechanisms which increase productivity of female scientists. Addressing this gender equity gap in global health is essential to achieving the Sustainable Development Goals (SDGs). Program to sensitize men on gender equality and prevention of sexual exploitation, abuse, and harassment (PRSEAH) are highly needed in professional and academic milieu. Provision of childcare in professional milieux, call for publications, grants, mentorship, studentships, and scholarships opportunities for women in science and African institutions can contribute to closing the gender gap in global health research.

## Supporting information

S1 Data (XLSX)
